# A Pilot Study on Data-Driven Adaptive Deep Brain Stimulation in Chronically Implanted Essential Tremor Patients

**DOI:** 10.3389/fnhum.2020.541625

**Published:** 2020-11-05

**Authors:** Sebastián Castaño-Candamil, Benjamin I. Ferleger, Andrew Haddock, Sarah S. Cooper, Jeffrey Herron, Andrew Ko, Howard. J. Chizeck, Michael Tangermann

**Affiliations:** ^1^Brain State Decoding Lab, Department of Computer Science, BrainLinks-BrainTools Cluster of Excellence, University of Freiburg, Freiburg im Breisgau, Germany; ^2^BioRobotics Lab, Department of Electrical and Computer Engineering, University of Washington, Seattle, WA, United States; ^3^Department of Neurological Surgery, University of Washington Medical Center, Seattle, WA, United States; ^4^Autonomous Intelligent Systems, Department of Computer Science, University of Freiburg, Freiburg im Breisgau, Germany; ^5^Artificial Cognitive Systems Lab, Artificial Intelligence Department, Faculty of Social Sciences, Donders Institute for Brain, Cognition and Behaviour, Radboud University, Nijmegen, Netherlands

**Keywords:** deep brain stimulation, neural decoding, essential tremor, machine learning, adaptive deep brain stimulation, closed-loop deep brain stimulation

## Abstract

Deep brain stimulation (DBS) is an established therapy for Parkinson's disease (PD) and essential-tremor (ET). In adaptive DBS (aDBS) systems, online tuning of stimulation parameters as a function of neural signals may improve treatment efficacy and reduce side-effects. State-of-the-art aDBS systems use symptom surrogates derived from neural signals—so-called neural markers (NMs)—defined on the patient-group level, and control strategies assuming stationarity of symptoms and NMs. We aim at improving these aDBS systems with (1) a data-driven approach for identifying patient- and session-specific NMs and (2) a control strategy coping with short-term non-stationary dynamics. The two building blocks are implemented as follows: (1) The data-driven NMs are based on a machine learning model estimating tremor intensity from electrocorticographic signals. (2) The control strategy accounts for local variability of tremor statistics. Our study with three chronically implanted ET patients amounted to five online sessions. Tremor quantified from accelerometer data shows that symptom suppression is at least equivalent to that of a continuous DBS strategy in 3 out-of 4 online tests, while considerably reducing net stimulation (at least 24%). In the remaining online test, symptom suppression was not significantly different from either the continuous strategy or the no treatment condition. We introduce a novel aDBS system for ET. It is the first aDBS system based on (1) a machine learning model to identify session-specific NMs, and (2) a control strategy coping with short-term non-stationary dynamics. We show the suitability of our aDBS approach for ET, which opens the door to its further study in a larger patient population.

## 1. Introduction

Deep brain stimulation (DBS) is an established clinical treatment for refractory stages of Parkinson's disease (PD), dystonia, and essential tremor (ET) (Krauss et al., 2004; Rodriguez-Oroz et al., 2005; Baizabal-Carvallo et al., 2014). In a standard clinical context, DBS parameters (as amplitude, frequency, pulse width, and electric field shape) are periodically determined by a trained expert for each patient. This recurring yet infrequent adaptation, accounts for post-surgical transient states and disease progression. However, it is insufficient for adapting to behavioral contexts and neurophysiological changes occurring on much shorter timescales. Furthermore, patients undergoing such continuous DBS (cDBS) therapy are prone not only to chronic motor and neuropsychiatric side-effects like speech disorders, dysarthria, depression, and emotional disinhibition (Bin-Mahfoodh et al., 2003; Appleby et al., 2007; Ondo et al., 2007; Witt et al., 2008, 2012; Fakhar et al., 2013; Castrioto et al., 2014; Little et al., 2016), but also to transient side-effects, including paresthesia, other speech disturbances, and gait ataxia (Kuncel et al., 2006; Appleby et al., 2007; Aldridge et al., 2016; Reich et al., 2016).

### 1.1. Closed-Loop Adaptive DBS

As an alternative to cDBS strategies, adaptive DBS (aDBS) systems use motor state surrogates to provide an online adaptation of DBS parameters. Such strategies decrease stimulation when it is not required, and thus may ameliorate DBS-induced side-effects (Little et al., 2013, 2016; Khobragade et al., 2015).

#### 1.1.1. Surrogates of Motor Performance

A key component of an aDBS system is a reliable motor state estimate, which can be quantified using inertial measurement units (IMU) or surface electromyography (Graupe et al., 2010; Herron et al., 2016). Alternatively, motor state surrogates can be extracted from brain signals, thus disregarding the necessity of external sensors (Little and Brown, 2012; Hoang et al., 2017; Panov et al., 2017). These motor state surrogates, termed neural markers (NMs), can be measured from local field potentials (LFP) of subcortical (Little et al., 2013; Priori et al., 2013) or cortical areas (Whitmer et al., 2012; Cao et al., 2017; Swann et al., 2018). A well-known example of NMs extracted from LFPs is the power of the beta-band (12–30 Hz), which—despite unclear causal relation and action mechanisms—is correlated with PD symptoms, such as bradykinesia and rigidity (Kühn et al., 2008, 2009; Whitmer et al., 2012; Blumenfeld and Brontë-Stewart, 2015; Neumann et al., 2017). Likewise, cortical band-power features have also been found to correlate with motor symptoms' severity in PD and ET (Weiss et al., 2015; Kondylis et al., 2016). The aforementioned studies follow a top-down approach for the identification of NMs by following *a priori* pathophysiological group-level knowledge about the disorder. While these surrogates facilitate the understanding of underlying neural dysfunctions, their informative value for controlling an aDBS system may be limited when it comes to an individual patient, because the heterogeneous phenotype of the diseases indicates that a global NM suited for all patients may not exist (Johnson et al., 2016). Such an NM seems even more elusive in a more semiologically complex disease, as PD, where research has been focused on symptom-wise NMs identification.

In contrast to top-down approaches used in the field of brain-computer interfaces (BCI) can be used to determine subject-specific NMs using machine learning (Blankertz et al., 2011; Tangermann et al., 2012; Neumann et al., 2019), thus improving motor state characterization of individual users (Meinel et al., 2016). Initial work in this direction has been presented by Connolly et al. (2015), who implemented machine learning methods to decode stages of PD in an animal model based on band-power and cross-frequency features. In more recent studies, Tan et al. (2019) and Yao et al. (2020) have argued in favor of a bottom-up approach for identification of NMs and discussed the implications that this may have on an aDBS system, however, their study was only implemented offline and thus, the suitability of such approach in a real scenario remains an open issue.

#### 1.1.2. Strategies of Closed-Loop Control for aDBS

Pioneering studies of aDBS for PD animal models utilized control strategies triggered by action potentials in the motor cortex or internal globus pallidus (Rosin et al., 2011). Later studies in human patients implemented uni-dimensional power-band features driving threshold-based controllers, yielding symptom suppression comparable to cDBS strategies, while having a significantly shorter effective stimulation time, as shown by Little et al. (2013, 2015). Likewise, Rosa et al. implemented a proportional control strategy based on the same oscillatory NMs, obtaining similar results in terms of symptom suppression and reduced net stimulation (Rosa et al., 2015). These studies stand out among the first approaches on aDBS systems for humans. In more recent contributions, Velisar et al. (2019) have improved upon them by utilizing fixed dual-threshold control implementing hysteresis which accounts for fast variations in the control signal.

These threshold-based and proportional control strategies generally disregard any state transition information or the temporal evolution of the symptoms and of the corresponding NMs, since the next control signal is determined based on just a single NM state measurement (the NM at the current time point). However, the temporal history of the NMs might contribute important information. For example, several authors have suggested temporal dynamics of beta-band power embedded in beta-burst characterization as potential source of dynamics-aware information (Tinkhauser et al., 2017; Moraud et al., 2018; Piña-Fuentes et al., 2019). Likewise, dynamics-aware control strategies have also been explored. For example, model predictive control for ET in an aDBS system based on IMU information (Haddock et al., 2017), coordinated-reset in PD patients and animal models (Adamchic et al., 2014; Wang et al., 2016), phase-dependent burst stimulation (Cagnan et al., 2016), or context-triggered strategies based on event-related desynchronization (Herron et al., 2017). These studies are an important indication for considering patient-specific temporal dynamics for control of aDBS systems.

### 1.2. Developing aDBS Systems for ET Patients

Developing novel aDBS systems is a challenging endeavor. For example in PD, the characterization of robust NMs by itself is a difficult task, mainly due to PD's phenotype hetereogenity and the difficulty of measuring axial symptoms and their delayed suppression upon DBS. Furthermore, the temporal dynamics in PD are non-trivial due to the DBS *washout*—a decaying clinical effect of DBS therapy observed after stimulation withdrawal—which affects different symptoms at different timescales (Cooper et al., 2013). In contrast, ET has several characteristics that renders it a simpler scenario for aDBS development, compared to PD. Notably, ET symptoms are generally restricted to kinetic and postural tremor, and the DBS washout effect is negligible. Finally, as the prevalence of ET is significantly greater, it is easier to investigate: a recent meta-study found that ET affects nearly 5% of the population over 65, compared to <2 % of the same demographic diagnosed with PD (Alves et al., 2008; Louis and Ferreira, 2010).

With our contribution, we present a proof-of-concept study of a novel closed-loop aDBS system with model-free control. To the best of our knowledge, it is the first system that implements (1) characterization of NMs based on machine learning and (2) a dynamics-aware control. As such, it addresses the major challenges found in aDBS. We provide results for three ET patients, totaling five experimental sessions, demonstrating the feasibility of our approach.

## 2. Methods

The proposed aDBS system is grounded on two main functional building blocks: (a) the estimation of ongoing tremor intensity based on individual spectral features extracted from ECoG signals, processed by a machine learning algorithm *(in section* *2.1* *NM identification based on machine learning methods)*; and (b) a model-free control strategy, that adapts the stimulation amplitude based on temporally local statistics of tremor prediction *(in section* *2.2* *control signal generation robust to non-stationary dynamics)*. In the following subsections, we will describe both functional building blocks and the specific methods used to implement them^1^. At the end of the section, a brief description of the Fahn-Tolosa-Marin (FTM) rating scale is provided, which is a clinical assessment tool that characterizes tremor intensity in patients and which we would also use for supporting the assessment of tremor.

### 2.1. NM Identification Based on Machine Learning Methods

The appearance of ET has been linked to dysfunctions in the cortico-thalamic-cerebellar loop. Specifically, anomalies in the connectivity and band-power activity of the motor cortex have been identified as physiological surrogates of the disease (Raethjen and Deuschl, 2012; van Wijk et al., 2012; Neely et al., 2014). Therefore, we propose to use the band-power of ECoG signals recorded from the primary motor cortex (M1) as information source to learn patient-specific NMs for the proposed data-driven tremor estimation.

Let $y\in {\mathbb{R}}^{N_{e}}$ be a vector containing average tremor intensity measured at *N*_*e*_ time windows, as characterized from an IMU. We propose to find a linear projection vector $w\in {\mathbb{R}}^{N_{f}+1}$, where *N*_*f*_ is the number of frequency bins of the ECoG signal, such that

(1) $$\begin{array}{r} \hat{y}=w^{𝖳}X \end{array}$$

with $\hat{y}\in {\mathbb{R}}^{N_{e}}$ denoting the predicted tremor intensity at *N*_*e*_ time windows, and $X\in {\mathbb{R}}^{N_{f}+1\times N_{e}}$ a matrix containing the spectral power of selected frequency bins computed from *N*_*e*_ time windows recorded from an ECoG electrode placed over M1, and a row containing only ones, for bias estimation. Tremor intensity ***y*** is an autocorrelated process since contiguous time points are not necessarily independent; however, for the sake of simplicity in our proof-of-concept system, we assume that the measurements of ***y*** have sufficient temporal distance such that the samples are independent and identically distributed. Under this assumption, the weights ***w*** can be estimated by solving the optimization problem ${\text{arg min}}_{w}∥y-\hat{y}{∥}^{2}$.

This ordinary least mean square regression problem can be solved analytically, resulting in a weight vector ***w*** = (***XX***^𝖳^)^−1^***Xy***.

### 2.2. Control Signal Generation Robust to Non-stationary Dynamics

In the closed-loop study by Little et al. (2013), thresholds on NMs to switch DBS on or off had been determined manually. Similarly, the proportional control strategy by Rosa et al. (2015) uses pre-estimated band-power ranges to determine a linear mapping to DBS amplitude. These approaches were successful (even in experiments involving freely moving PD patients) and are referents in the field.

Those fixed mappings between observed NMs and amplitude, however, presuppose the underlying neural system as a stationary process. Nonetheless, this assumption is problematic in aDBS: The dynamics of band power NMs are context-dependent and change upon, e.g., sitting, walking, or during transitory movement states (Bulea et al., 2014; Haddock et al., 2017). In addition, they are co-modulated by other processes, such as the circadian rhythm or medication intake (Pollok et al., 2012). Therefore, we propose a time-varying mapping of $\hat{y}$ to the DBS-amplitude, based on local *high* and *low* tremor intensity states, derived from moving statistics of the estimated tremor. Specifically, we define an increase or decrease in DBS amplitude Δ*u* by

(2) $$\begin{array}{l} \Delta u=\{\begin{array}{ll} u_{i}, & \text{if}\;\hat{y}>\delta_{h}^{t}\\ u_{d}, & \text{if}\;\hat{y}<\delta_{l}^{t}\\ 0, & \text{otherwise} \end{array} \end{array}$$

where $u_{i}\in {\mathbb{R}}^{+}$ and $u_{d}\in {\mathbb{R}}^{-}$ are scalars that respectively indicate an increase or decrease in stimulation amplitude, and $\delta_{h}^{t},\delta_{l}^{t}\in {\mathbb{R}}^{+}$ are the corresponding time-varying thresholds at time point *t*.

We use the Bollinger bands method (Bollinger, 2001) to compute $\delta_{h}^{t}$ and $\delta_{l}^{t}$. It is widely used in financial analysis for detecting trends in assets pricing, characterizing relative high and low states. In our case, the same principle is used to detect whether the current tremor estimation delivered a relative *high* or *low* intensity state, based on a short term history of the estimated tremor $\hat{y}$. Specifically, $\delta_{h}^{t}=a_{N}\left({\hat{y}}_{t}\right)+Kstd_{N}\left({\hat{y}}_{t}\right)$ and $\delta_{l}^{t}=a_{N}\left({\hat{y}}_{t}\right)-Kstd_{N}\left({\hat{y}}_{t}\right)$, where *K* ∈ ℝ^+^ is a scaling constant, $a_{N}\left({\hat{y}}_{t}\right)$ is the moving average of $\hat{y}$ computed in the time interval [*t*−*N, t*], and $std_{N}\left({\hat{y}}_{t}\right)$ defines the standard deviation of $\hat{y}$ in the same period of time.

### 2.3. Binary and Graded aDBS

We propose two approaches for determining the control signals *u*_*i*_ and *u*_*d*_, inspired by the threshold-based aDBS and proportional aDBS systems used in Little et al. (2013), Rosa et al. (2015), and Velisar et al. (2019):

In the data-driven *binary aDBS* (**b-aDBS**), only DBS “on” and “off” states are considered, i.e., *u*_*i*_ = −*u*_*d*_ = *A*_*cDBS*_, where *A*_*cDBS*_ corresponds to the patient-specific DBS amplitude optimized by a trained expert for clinical cDBS therapy.

In the data-driven *graded aDBS* (**g-aDBS**), a granular control of the DBS amplitude is provided by *u*_*i*_ = −*u*_*d*_ = 0.5*V*, which is the minimum voltage change Δ*u* implementable in the available hardware platform.

In both cases, the stimulation amplitude is restricted to the interval [0, *A*_*cDBS*_].

### 2.4. Clinical Assessment of Tremor

In a standard clinical context, the FTM is used for assessing the tremor intensity in ET patients and the corresponding efficacy of DBS or standard pharmacological treatment. We will use the FTM scale as one of the assessment criteria for our developed systems. The FTM assessment is divided into several items that evaluate axial symptoms, motor activities (such as drawing or water pouring), as well as tremor intensity in specific limbs. These items are scored with integer numbers from 0 (no tremor), up to 4 (tremor amplitude >2 cm). For more details about the FTM scoring system, we refer the reader to the original publication (Fahn et al., 1993).

## 3. Experimental Setup

### 3.1. Patients

This study was conducted under supervision of the University of Washington Institutional Review Board following the set of ethical principles outlined in the Declaration of Helsinki regarding human experimentation. Experiments were conducted in five sessions performed with three right-handed patients diagnosed with ET: one session with patient 1 ($S_{1}^{1}$), and two sessions each with patient 2 ($S_{2}^{1}$, $S_{2}^{2}$), and patient 3 ($S_{3}^{1}$, $S_{3}^{2}$). All patients were unilaterally implanted with DBS electrodes in the left ventral intermediate nucleus and with an four-electrode linear ECoG strip (re-purposed Medtronic Resume II spinal cord stimulation electrode with four contacts) over the hand area of the left M1. The ECoG strip was positioned using steady state evoked potentials obtained from the median nerve to identify the hand sensorimotor cortex. The canonical ventral intermediate nucleus coordinates are targeted based on the anterior and posterior commissural points (AC/PC) rectification of MRI and corrections based on patient anatomy. X is left-right, Y is anterior-posterior, and Z is superior-inferior. Canonical target is: X = 0.55 × AC/PC distance lateral to midline; Y = 0.25 × AC/PC distance posterior to mid-commissural point (half the distance between AC and PC); Z is at a plane defined by the line between AC and PC. Additionally, the location of the internal capsule and the width of the third ventricle is examined. The electrode is positioned at least 3 mm from the border of the internal capsule, which is usually about 10.5 mm + 1/2 the width of the third ventricle, roughly corresponds the X as calculated above. DBS lead and ECoG strip location were confirmed with post-operative CT scan.

Signal acquisition and DBS was performed with the implantable Medtronic Activa PC + S, an investigational neurostimulator approved for use in this research through both an FDA investigational device exemption. The ECoG recording electrode configuration was determined in a different study as the most effective for achieving volitional control of DBS, with the same patient population here presented (Houston et al., 2018).

Excepting stimulation amplitude, DBS parameters were kept unchanged from clinical decisions, and thus vary between subjects, as found in Table 1. The same table shows the time elapsed between implantation surgery and execution of the corresponding experimental session, and amount of data collected per session.

**Table 1:** Information about experimental sessions: Months since implantation (MSI), therapeutical cDBS parameters (amplitude, frequency, and pulse width), total amount of *rest* and *posture* trials, and the resulting time segments utilized for training the tremor decoding model.

	**MSI**	**cDBS parameters**	**Rest-posture trials**	***N*_*e*_**
$S_{1}^{1}$	22	2.5 V, 140 Hz, 90 μs	22	220
$S_{2}^{1}$	13	3.9 V, 130 Hz, 90 μs	20	200
$S_{2}^{2}$	16	4.1 V, 130 Hz, 90 μs	20	200
$S_{3}^{1}$	5	2.9 V, 140 Hz, 60 μs	20	200
$S_{3}^{2}$	12	3.1 V, 140 Hz, 60 μs	30	83

### 3.2. Session Design

**Figure 1:**
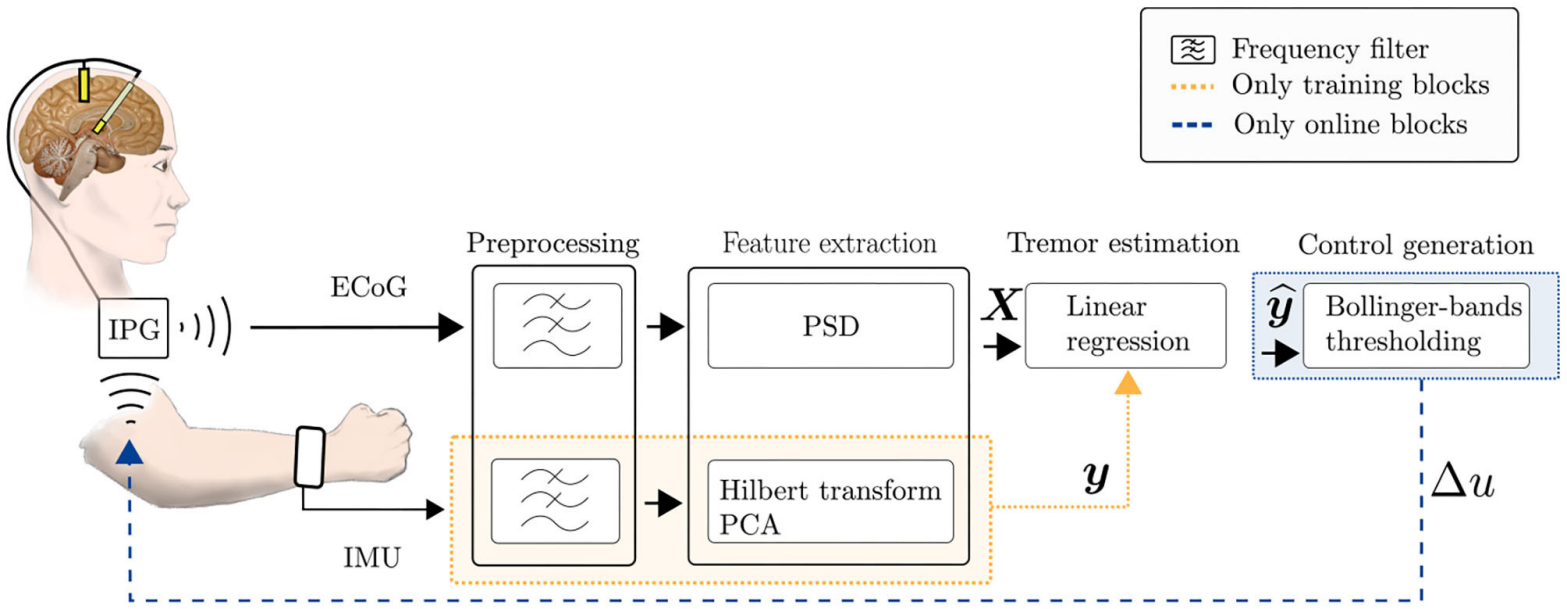
Scheme of the implemented data-driven aDBS system, for training and online stages. Firstly, ECoG data and IMU data are collected for training the tremor estimation model. During the online stage, the tremor estimated by the trained linear regression model of Equation (1) is used for generating the DBS control signal in Equation (2).

Figure 1 shows an overview of the implemented system and its individual components, as described in the previous section. In the following, the training and testing stages of the system will be explained.

#### 3.2.1. Training Data Collection

Training data was collected during a cDBS parameter optimization procedure carried out for a parallel study (please refer to Haddock et al., 2018 for further information). Patients sat at rest in a chair with hands in their laps; for each *trial*, the experimenter prompted patients to move the dominant hand to a patient-specific tremor-eliciting *posture*, where it was held during a 10 s interval, followed by a 30 s *rest* period. For the tremor-eliciting *posture*, patients were instructed to conduct the “arms extended” and “wing-beating” postural tests of the TETRAS test (Elble et al., 2016). If these tests did not generate sufficient tremor, patients were asked to hold a posture they knew to be especially troublesome while untreated. Specifically, for *S*_1_ and *S*_3_, the “wings” posture was most effective, while for *S*_2_ imitating the act of holding a screwdriver to a fixed point was most effective.

Even though different DBS configurations were applied throughout the stage, only trials performed during DBS-off were used as training data. From these trials, only *posture* segments were used. Restricting our analysis to the posture condition only is not a useful distinction in a clinical aDBS. However, for this pilot study, we want to prioritize NMs that do not represent voluntary movements. In a scenario where one would consider both, posture and rest conditions, then the derived labels *y* would be structured as two large clusters of tremor activity corresponding to these conditions. The tremor would vary within each of them, but the largest variation might be between them. So if both conditions are considered, any NM that we extract to capture variations in tremor, might be related to tremor itself (and would be an appropriate feedback signal for the aDBS system), or might be related only to posture and rest conditions but unrelated to any pathology. The latter, of course, would be unsuitable as a feedback signal for the control system because the tremor label would have acted just as a label of rest/posture conditions, and not as a label of pathological tremor.

The total amount of rest-posture trials collected during this stage can be found in Table 1.

#### 3.2.2. Online Stage—Posture Prompt

Following the training run, the b-aDBS and g-aDBS approaches were applied online. Analog to the training stage, a computer screen prompted patients to remain at *rest* during 20 s, and then to hold that same patient-specific tremor-inducing *posture* during 30 s before going back to the rest position. In total, 12 rest-posture trials were collected for each controller type during this online stage.

#### 3.2.3. Online Stage—Clinical Assessment

In the final phase of the experimental sessions, the clinical efficacy of the aDBS strategies was compared to cDBS and DBS off, using parts A and B of the FTM scale (Fahn et al., 1993). The FTM tests were captured on camera and the videos were evaluated offline by two blinded clinical experts. Due to time constraints, the FTM assessment could not be performed for all aDBS conditions. As previous studies indicate a similar clinical outcome of binary aDBS and cDBS (Herron et al., 2016), we decided to perform the video recordings for g-aDBS only and not for b-aDBS. Due to logistic constraints, this clinical assessment was performed only for sessions $S_{2}^{1}$, $S_{3}^{1}$, and $S_{3}^{2}$.

### 3.3. Signal Acquisition and Pre-processing

LFP data was recorded from a single ECoG channel with a sampling rate of 422 Hz. Data was streamed at 400 ms intervals from the Activa PC + S unit to an RF receiver connected through USB to an external computer, where all relevant computation was conducted. Angular velocity and linear acceleration were recorded in three orthogonal spatial directions at 100 Hz using the IMU contained in an *LG G smartwatch* fastened onto the subject's right wrist, resulting in six IMU channels. Since ECoG and IMU data were acquired with different systems at different sampling rates, signals had to be aligned with respect to a common timestamp. This alignment was updated with the beginning of each rest-posture trial. IMU signals were band-pass filtered with a 5-th order butterworth filter in the band corresponding to pathological tremor, i.e., [4 − 7] Hz. This frequency band was fixed for all sessions, however, we confirmed in the offline analysis that the pathological tremor for all patients was found in this frequency band (not shown).

The aligned IMU and ECoG signals were segmented into ten continuous, non-overlapping 1 s epochs per trial, such that up to 220 epochs were available per patient. An artifact rejection stage was applied, removing segments containing ECoG signals with a peak-to-peak amplitude ≥ 3 mV. For training data, segments belonging to the transient stages of movement—i.e., transitions between rest and posture conditions, and vice versa—were removed from the analysis. Such segments were identified by detecting epochs where any IMU channel showed a standard deviation of more than five times the IMU channel-wise average standard deviation across epochs, in the tremor frequency band.

Table 1 shows the final number of epochs *N*_*e*_ available for training of the tremor decoding model. Note that for $S_{3}^{2}$, ~72 % of the epochs had to be rejected due to artifacts and inconsistent patient's pose during the posture condition. For the rest of the sessions, all data collected during *posture* was utilized.

### 3.4. Signal Characterization

#### 3.4.1. Tremor Characterization

For obtaining tremor labels ***y***, the envelope of the band-pass filtered IMU signals was extracted as the magnitude of the Hilbert transform. Average channel-wise IMU power was computed for each of the epochs by averaging the envelope across time. The resulting *N*_*e*_ × 6 matrix was subsequently standardized along the first dimension. Finally, principal component analysis (PCA) was performed and the signals were projected onto the principal component associated with the largest eigenvalue of the corresponding decomposition, thus yielding an unidimensional representation of tremor intensity, used as the ground truth label ***y*** for training and validating the regression model in Equation 1.

#### 3.4.2. Neural Signal Characterization

For extracting neural features, the power spectral density (PSD) of the ECoG signal was computed for each epoch using the Welch method based on the fast Fourier transform computed with 256 coefficients. Only spectral features in the interval [3−25] Hz were considered for further analysis, resulting in fourteen 1.56 Hz-wide frequency bins. The motivation for limiting the analysis to this frequency band lies on the spectral properties of stimulation and muscle artifacts, which are sometimes detectable in the >25 Hz rhythms. Even though ECoG signals are rather robust to muscle artifacts compared to non-invasive recordings, such as electroencephalographic signals, the pilot character of our study called for a more conservative approach to the experimental setup, which further enforced this design decision. However, we think that limiting the spectral analysis to this band does not erode the significance of results obtained, since NMs found in the literature are also typically found in this frequency range.

### 3.5. Training of the Tremor Decoding Model

A subset of the 14 ECoG spectral features were used to construct a patient- and session specific training data set ***X***. The subset was determined using a top-down feature selection procedure, where the full spectral feature set was iteratively pruned until the regression model's performance ceased to increase. In each iteration, the least important feature, as characterized by the corresponding weight in ***w*** was removed and the linear model was re-trained with the remaining features. Using a chronological 5-fold crossvalidation procedure (without sample shuffling), the decoding performance was assessed using the Pearson correlation coefficient ρ between ***y*** and $\hat{y}$. If a performance increase with respect to the previous iteration was observed, the pruned feature was left out and the iterative procedure was continued. Otherwise, the pruning stopped.

### 3.6. Control Signal Generation

The moving statistics determining $\delta_{h}^{t}$ and $\delta_{l}^{t}$ were computed using a time window of 20 s and a standard deviation scaling constant *K* = 2. These hyperparameters were not optimized per patient but fixed prior to the study. A control signal was issued according to the rules defined in sections 2.2 and 2.3 every time a new data package was available, i.e., every 400 ms.

## 4. Results

### 4.1. Spectral Feature Relevance

**Figure 2:**
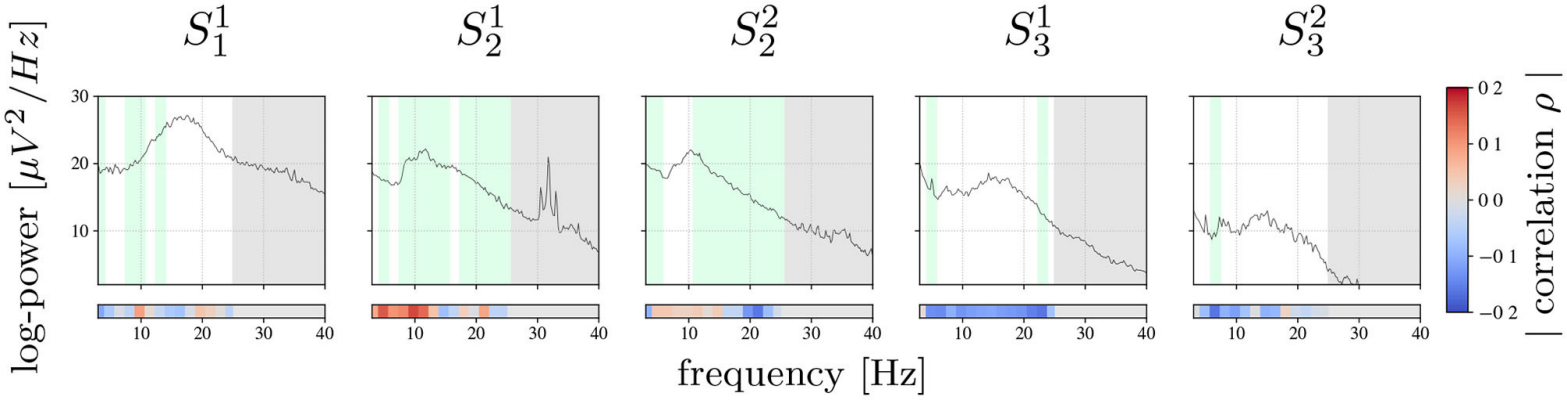
Session-wise averaged PSD computed for training data. Bars on the bottom show the Pearson correlation achieved between each frequency bin and true tremor labels ***y***. Highlighted in green are the frequency bins selected by the feature selection algorithm. Marked with gray are frequency bins that were not used for the analysis.

Figure 2 shows the average PSD calculated for training data and the corresponding correlation ρ between band-power in each frequency bin of the ECoG signals and labels ***y***. Furthermore, the features selected by the top-down feature selection procedure are highlighted in green. The spectra show a high inter-patient variability: for patient 1, the spectrum is characterized by a prominent beta peak, similar to patient 3, whereas patient 2 is dominated by an alpha-band component. There is also a pronounced within patient variability across sessions in terms of the absolute spectral power. The frequency band of prominent spectral peaks, however, is constant across sessions within subject, i.e., alpha-band for patient 2 and beta-band for patient 3.

Power band features revealing the strongest correlation with tremor intensity vary considerably between patients: for $S_{1}^{1}$, $S_{3}^{2}$, and $S_{2}^{1}$ the frequency bins with the strongest correlation are in the alpha- and theta-band, whereas for $S_{2}^{2}$ and $S_{3}^{1}$ the most informative frequency bins are found beyond 10 Hz, mainly in the higher beta-band.

In contrast, features selected for inclusion in the tremor prediction model were found all across the spectrum analyzed. The absence of spectrally compact features may be explained by the high redundancy of neighboring frequency bins and as the feature selection procedure typically selects one only out of multiple bins with redundant information.

**Figure 3:**
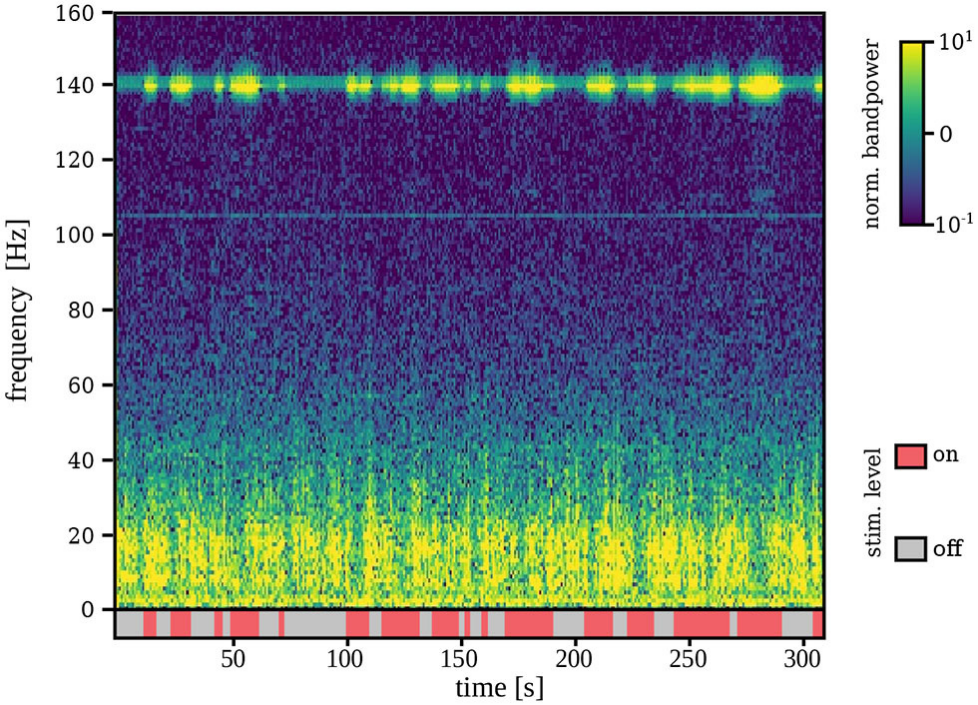
Time-frequency representation of the ECoG signal during stimulation on and off for a representative example (*S*_1_). Although stimulation artifacts are clearly visible, signal is not saturated and lower-frequency components are measurable even during stimulation on.

Figure 3 shows a representative example of the robustness of the spectral features used for tremor decoding under different DBS conditions. Specifically, it depicts a segment of ECoG data recorded during the online phase of session *S*_1_. The stimulation artifact is clearly visible, nevertheless, it does not impede measurement of low-frequency components due to saturation of the amplifiers or sub-harmonics of the stimulation.

### 4.2. Tremor Estimation Accuracy

Table 2 shows the average Pearson correlation coefficient between estimated and true tremor intensity^2^. They indicate the tremor decoding accuracy during training and online stages. As a baseline, the average correlation between the theta-band power and true tremor intensity is also considered, which is a well-known NM for ET stemming from group-level studies (Kane et al., 2009). All scores derived from the training stage were computed using a 5-fold crossvalidation without shuffling. Statistical significance was defined at an uncorrected *p* < 0.02 for the probability that the score was obtained by chance under a bootstrapping procedure for 1,000 label shuffles.

**Table 2:** Average linear correlations between estimated and true tremor intensities.

	**Training stage**			**Online stage**	
	**Theta-power (4–7 Hz)**	**Data-driven**	**Informative band**	**b-aDBS**	**g-aDBS**
$S_{1}^{1}$	−0.06	0.39^*^	alpha	n/a	n/a
$S_{2}^{1}$	0.22^*^	0.21^*^	theta/alpha	−0.15	−0.10
$S_{2}^{2}$	0.16	0.22^*^	beta	0.05	0.12^*^
$S_{3}^{1}$	−0.14^*^	0.29^*^	theta/beta	0.29^*^	0.35^*^
$S_{3}^{2}$	−0.18	0.05	theta	n/a	0.20^*^

Statistical significance (indicated by ^*^) is defined at an uncorrected p < 0.02 obtained with a bootstrapping procedure with 1,000 label shuffles. Additionally, column informative band shows the frequency band with the largest correlations with tremor intensity, according to Figure 2.

**Figure 4:**
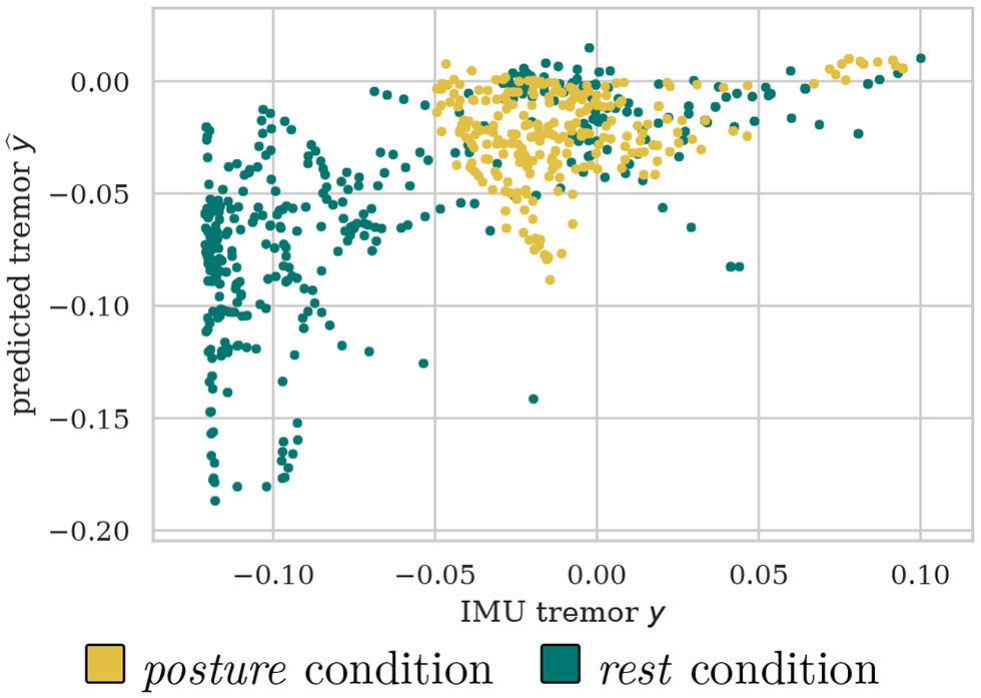
Example scatter plot for $S_{3}^{1}$ of predicted vs. measured tremor intensities discriminated between *posture* and *rest* conditions.

It can be observed that the proposed data-driven tremor decoding model achieved a significant correlation in four out of the five sessions for the training stage. During the online stage, in three out of four sessions conducted, statistically significant decoding performance was obtained. Overall, the decoding performance of the data-driven model is superior to the fixed theta-band power, however, the correlation achieved is weak in all sessions analyzed. Figure 4 shows a representative example of measured vs. estimated tremor, for session $S_{3}^{1}$.

### 4.3. Control Signals Distribution

**Figure 5:**
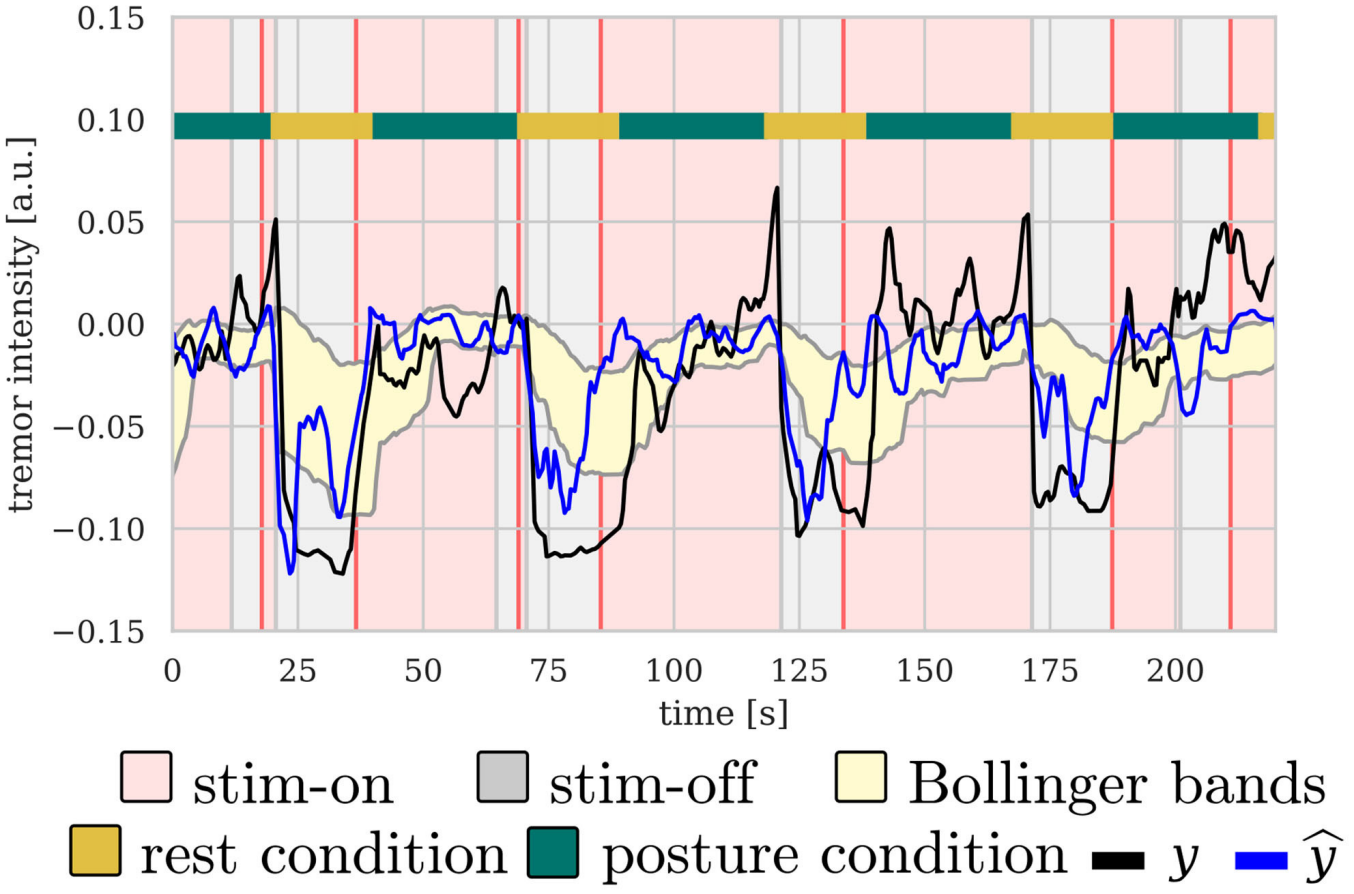
Illustrative example of the control signal for b-aDBS computed during the online stage of $S_{3}^{1}$. There is a clear correlation between *posture* condition and tremor intensity, both predicted and measured, thus, triggering stimulation mainly during *posture* condition.

Figure 5 shows an illustrative example of the control signal, including the Bollinger bands, as well as measured and predicted tremor. As expected, predicted and measured tremor intensity increases during the *posture* condition, triggering the stimulation most of the times.

**Figure 6:**
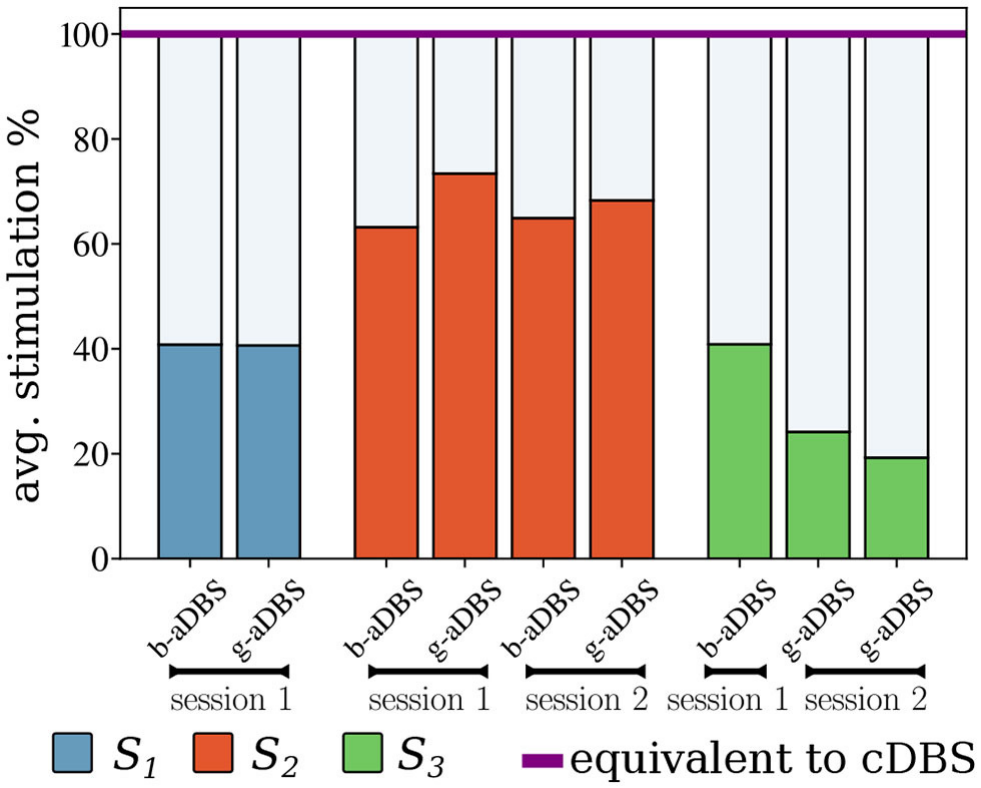
Average time stimulated relative to the stimulation using the equivalent cDBS strategy.

Figure 6 shows the average stimulation time during the online stages, compared to the equivalent cDBS strategy. It can be observed that for all types of controllers, the average time stimulated was considerably lower than that of the cDBS strategy. Furthermore, there is an indication for low intra-subject variability of average stimulation, whereas inter-subject variability might be larger. The total stimulation duration of b-aDBS and g-aDBS strategies was similar within patients.

### 4.4. Tremor Suppression in Online Stage

**Figure 7:**
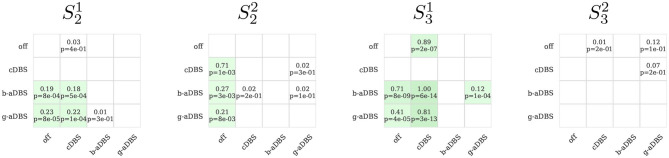
Pairwise comparison of tremor intensity under different stimulation strategies during the online stage (only *posture* segments). In green, it is highlighted if the method in the y-axis achieved a greater tremor suppression compared to the corresponding method in th x-axis. In each box, top number indicates the effect size and bottom number the corresponding *p*-value obtained with the Mann-Whitney rank test, comparing the method on the y-axis against the corresponding method in the x-axis. Only positive effect sizes are shown. Green boxes indicate uncorrected *p* < 0.05. For the comparison, 1 s windows were used, extracted from the 30 s posture intervals of the 12 trials executed for the online stage.

Figure 7 compares tremor intensity suppression (1 − ***y***) between all stimulation strategies during online stages, under *posture* condition. Each box shows at the standardized mean difference of the pairwise comparison (top) and the *p*-values of the corresponding Mann-Whitney rank test (bottom). If standardized mean difference is negative, no values are shown. For $S_{3}^{2}$, no significant difference was established among all considered conditions, whereas for $S_{2}^{1}$ and $S_{3}^{1}$, adaptive strategies achieved superior tremor suppression than cDBS and improved upon DBS-off. For $S_{2}^{2}$, all stimulation strategies improved upon DBS-off, but no differences could be found among them. Even though, we expected aDBS to perform as good as cDBS, and better than DBS-off, cDBS only performed better than DBS-off in $S_{2}^{2}$, and even worse in $S_{3}^{1}$, suggesting a suboptimal setting of therapeutic parameters in cDBS. Overall, all significant differences reflect a small to medium size effect.

### 4.5. Clinical Tremor Assessment

Table 3 shows the FTM scores averaged for both clinical raters. Axial scores reported here comprises the sum of face, tongue, head, and trunk tremor scores. The scores for left/right upper/lower tremor comprises the sum of scores obtained during rest, posture, and action (finger to nose and toe to finger). For subtests with a discrepancy between clinical raters >1 point, we marked the averaged value (*) and provided both individual scores in parenthesis.

**Table 3:** Averaged scores of parts A and B of the FTM assessment for sessions $S_{2}^{1}$, $S_{3}^{1}$, and $S_{3}^{2}$.

	$S_{2}^{1}$			$S_{3}^{1}$			$S_{3}^{2}$		
**Test**	**cDBS**	**DBS off**	**g-aDBS**	**cDBS**	**DBS off**	**g-aDBS**	**cDBS**	**DBS off**	**g-aDBS**
Axial	1.5	1.5	0	0.5	0	0	0.5	0	0
Speaking	1	0.5	1	0	0	0	0	0	0
Handwriting	0.5	0.5	0.5	0	2.5	0.5	0.5	1.5	1
Left upper	4.5	3.5	3.5	2* (1/3)	1* (0/2)	1.5	1	1* (0/2)	1.5
Left lower	0	0.5	0.5	0	0	0	0	0.5	0.5
Left drawing	3	3	3	1.5	1	1	1.5	1	1.5
Left pouring	3	3	3	0.5	0.5	0	0	0	0.5
Right upper	3	4* (5/3)	3* (4/2)	3.5	3	3	2.5* (4/1)	3	3
Right lower	0	0	0	0	0.5	0.5	0.5	0.5	0.5
Right drawing	1.5	1	1	2	2	1	1.5	2	1
Right pouring	1	1	1	1	1	0	0.5	1	1* (0/2)
Total	19	18.5	16.5	11	11.5	7.5	8.5	10.5	10.5

Scores marked with a * indicate a discrepancy of more than 1 point between the scores assigned by each of the clinicians, followed by the individual scores in parenthesis. Due to logistical constraints, FTM assessment is only available for g-aDBS.

Considering the total FTM score per session, the proposed g-aDBS strategy did not lead to a worse FTM score than DBS off in none of the three sessions. The g-aDBS system achieved at least a moderate symptom suppression in two out of the three online sessions analyzed ($S_{2}^{1}$ and $S_{3}^{1}$). In these two sessions, cDBS and DBS off did not perform significantly different, while the g-aDBS score improved moderately by 2 points for $S_{2}^{1}$ and markedly by 3.5 points for $S_{3}^{1}$. For $S_{3}^{2}$, g-aDBS did not improve the symptoms compared to DBS off, while standard treatment cDBS reached an improvement of 2 points, indicating a moderate tremor amelioration.

A closer look at the subtests of FTM reveals that at least one point of improvement (mild tremor amelioration) between g-aDBS and the baselines cDBS and DBS off were obtained for axial and upper lateral scores for $S_{2}^{1}$. For $S_{3}^{1}$, handwriting, drawing, and pouring liquid with the right hand were the sub-tests for which g-aDBS achieved a mild improvement. Interestingly for $S_{3}^{2}$, g-aDBS did not worsen any symptom by more than one point (mild worsening). However, it improved drawing with the right hand by one point, compared to DBS off. In the other sub-tests, differences were at most 0.5 points, which is within the expected fluctuations over the course of a day (Pulliam et al., 2014) and indicates a marginal effect upon symptoms.

The b-/g-aDBS strategies were driven by NMs for right hand tremor (location of the IMU) extracted during *posture*. Consequently, it is important to analyze the specific scores for this item of the FTM individually: clinical raters assessed right hand postural tremor under DBS off for all the sessions as either absent (FTM score 0) or *slight* (FTM score 1 meaning an amplitude of <0.5 cm). These low tremor ratings may also offer an explanation for the low size effects shown in Figure 7. Under cDBS, it was reported that in $S_{2}^{1}$ and $S_{3}^{2}$ tremor improved, while for $S_{3}^{1}$ no difference could be established. The evaluation of the g-aDBS strategy showed the same improvement as for cDBS, except for one of the clinical raters who stated that for $S_{2}^{1}$ tremor increased by 1 point to moderate (0.5–1 cm tremor amplitude).

## 5. Discussion

We have presented a proof-of-concept study demonstrating the suitability of data-driven closed-loop aDBS strategies for treating ET patients. Our proposed system is based on session-specific, data-driven NMs obtained by a machine learning model, and a model-free control strategy accounting for non-stationary dynamics of the controlled system.

### 5.1. Using Machine Learning for Data-Driven Decoding of Tremor

Using our data-driven approach, tremor intensity could be decoded from spectral information contained in M1 ECoG signals, yielding a correlation value ranging from 0.21 to 0.39. This is a significant improvement compared to tremor decoding using solely theta-band power. Using the latter, a significant decoding performance was achieved in only two sessions. It is noteworthy that in one of the two sessions where theta-band power was informative about tremor intensity, a negative correlation was found. This not only evinces the poor generalization of NMs motivated by top-down approaches, but also shows the ambiguity in their information content. One observation confirmed by the D Agostinos *K*^2^ test is that the kurtosis and skewness of tremor *y* and estimated tremor ŷ deviate from a Gaussian distribution. This calls for caution when using the Pearson correlation coefficient, as in our case. However, the absence of long tails and outliers, and the fact that only the relative differences in correlation are important in our approach, makes this chosen decoding performance score an acceptable selection.

Furthermore, our decoding approach demonstrates that informative features are present in power of frequency bins found in the range of [3 − 25] Hz and that the tremor estimation should not be limited to a single frequency band defined a priori. This result does not only confirm the necessity of data-driven NMs identification for ET, but also has important implications in the development of aDBS systems for more phenotypically heterogeneous disorders, such as PD, where patient and symptom specific characterization of the motor symptoms may improve aDBS even further.

We have also identified non-stationary dynamics contained in the NMs used. We have observed variations of global spectral features across sessions, as well as heterogeneity in the spectral feature information content, as described by the varying correlation scores between power in individual frequency bins and the tremor intensity, within patient, across sessions. Such variability in the feature information content and in tremor decoding performance within subject—for example $S_{3}^{1}$ and $S_{3}^{2}$—suggest an underlying mixture of processes that might correlate with tremor intensity, but that cannot be captured from spectral features extracted only from one contralateral ECoG channel in M1. Consequently, multimodal and multidimensional data-driven NMs should be explored.

### 5.2. Generation of Dynamics-Aware Control Signal

The model-free control strategy implemented in our system accounted for non-stationary dynamics of tremor estimation. Although, the number of patients included in the study is too small for a statistical analysis, the few sessions available indicated that accounting for non-stationary dynamics can allow to identify local tremor states. Their existence may explain symptom suppression achieved by our g-aDBS system in a wider variety of conditions during the FTM evaluation, compared to cDBS and DBS off.

Our control strategy does not account for non-stationary dynamics in the NMs space, but directly in the space where tremor estimation is found. However, different neural features may be governed by different non-stationary dynamics stemming from factors, such as the circadian rhythm, current physical activity, medication, and surgery-induced stun-effect. Therefore, accounting for non-stationary dynamics directly in the NM space might provide a more robust feedback signal. This should be subject to future studies, where a longer time horizon shall enable the study of multi-time scale dynamics as described above.

### 5.3. Clinical Assessment

From a clinical perspective, the g-aDBS strategy performed better than cDBS in two out of the three sessions assessed with the FTM scale. Unfortunately, one of the limiting factors in our study is that only the g-aDBS strategy, and not b-aDBS, was evaluated using the FTM scale. In general, the clinical evaluation of motor diseases, as PD and ET, requires a highly trained clinician and a lengthy assessment protocol. Such requirements play a major role in time-constrained situations as those encountered in typical experimental sessions.

An interesting observation regarding the FTM assessment under g-aDBS is that for $S_{3}^{1}$, the strongest symptom improvements were achieved for the right side of the body. Even though, the reduced number of sessions limits the interpretability of this observation, a possible explanation for this may be that the g-aDBS controller was triggered by NMs extracted from the left hemisphere, resulting in a right-sided biased symptom suppression. As a consequence, we suggest that NMs should be extracted bilaterally.

From the patients' perspective, they could clearly differentiate between no stimulation and active stimulation, but could not identify substantial differences between cDBS and g-aDBS. In b-aDBS, patients reported occasional paresthesias in their treated upper limb. This mainly occurred while stimulation was ramping up from 0 to the maximum amplitude due to the ramping rate required to keep b-aDBS effective (Meidahl et al., 2017).

### 5.4. Power Consumption Optimization

Our system achieved a reduction of at least 24% and as much as 80% of stimulation time. According to Khanna et al. (2015), the breakeven point of the Activa PC + S regarding power consumption in closed-loop mode is at a reduction of 6%. Therefore, our system allows a considerable reduction in power consumption well above this threshold. It is important to mention that modern systems, such as the Activa RC by Medtronic or Vercise by Boston Scientific, have rechargeable battery systems, where power consumption is not as critical as in older non-rechargeable systems. Another typical constraint when implementing aDBS in clinical grade systems is that the available platforms have low computational capacity, which limits the complexity of the algorithms that can be used. Fortunately, most computationally expensive parts of our system can be implemented by a fast Fourier transform (power spectrum estimation) and a linear projection (tremor estimation model). Both operations are relatively inexpensive and are easily implementable in simple embedded systems contained in modern DBS.

### 5.5. Limitations and Open Questions

#### 5.5.1. Clinical Open Questions

The greatest limiting factor of our current contribution is the small sample size and partially conflicting outcome regarding the efficacy of the clinical cDBS condition used as control. Specifically according to the FTM scale, cDBS only performed better than DBS-off in one session, suggesting that cDBS suffered suboptimal therapeutic parameter settings, which may also define a ceiling for the effect of aDBS. This calls for a larger clinical study, where the efficacy of the proposed system can be drawn as a statistically sounding conclusion.

Another important item is clinical safety of our approach. Even though our patients did not report any side effects during treatment with aDBS (besides transient paresthesias) and we think that our strategy does not represent any risk different from those encountered in existing aDBS strategies, the safety profile of our approach is still an open issue and should be further investigated with more patients.

#### 5.5.2. Technical Open Questions

From a technical point of view, there are also several open questions to consider. First, we limited the training segments to *posture* condition only, this allowed us to obtain a model that effectively decodes tremor intensity during tremor-inducing conditions and should not contain discriminative information about the posture itself or movement onset. If *rest* segments would have also been included, our model would potentially learn to decode the motor task (going from *rest* to *posture* and viceversa). Although, detection of movement onset may provide additional information for controlling the system (Herron et al., 2017; Tan et al., 2019), our main goal was to obtain an aDBS system relying on symptom surrogates. This design decision limits the generalization of our approach to other postural conditions, which should be subject of further investigation.

Our system can account for spectral fluctuation of a specific NM in short and long term, since the Bollinger-bands consider a history of its activity. A large contextual change (e.g., falling asleep), however, may render the chosen NM uninformative and would limit our approach. In this case, an adaptation of the decoder (i.e., using a different NM) will be necessary. This shall be subject to future studies.

Finally, limiting the features to spectral power of M1 signals might reduce the decoding power of the underlying machine learning model. To improve upon this limitation, future systems shall include more complex features, for example as used by Yao et al. (2020) in their most recent work.

## 6. Conclusions

Our contribution offers the first data-driven aDBS system based on machine learning methods, accounting for short-term non-stationary dynamics, and allowing online patient-specific optimization in DBS therapy. As outlook, we foresee the clinical validation of the novel strategies presented here and the development of more advanced decoding techniques and control strategies to tackle the open challenges regarding non-stationary dynamics present in diseases, such as PD.

## Data Availability Statement

The datasets generated for this study are available on request to the corresponding author.

## Ethics Statement

The studies involving human participants were reviewed and approved by University of Washington Institutional Review Board. The patients/participants provided their written informed consent to participate in this study.

## Author Contributions

SC-C, BF, HC, and MT conceived the methods and wrote the paper. SC-C and BF method implementation and contributed the analysis tools. SC-C, BF, SC, AH, and AK performed the experiments. SC-C, BF, SC, JH, AK, and MT analyzed the data. All authors contributed to the article and approved the submitted version.

## Conflict of Interest

The authors declare that the research was conducted in the absence of any commercial or financial relationships that could be construed as a potential conflict of interest.

## Footnotes

^1^All methods were implemented using the publicly available MNE python (Gramfort et al., 2013) and scikit-learn (Pedregosa et al., 2011).

^2^For $S_{1}^{1}$ no IMU data was available during the online stage and consequently, tremor decoding accuracy scores can not be reported. For $S_{3}^{2}$, no online stage was executed for b-aDBS due to time constraints.
